# Recent trends in the U.S. Behavioral and Social Sciences Research (BSSR) workforce

**DOI:** 10.1371/journal.pone.0170887

**Published:** 2017-02-06

**Authors:** Hyungjo Hur, Maryam A. Andalib, Julie A. Maurer, Joshua D. Hawley, Navid Ghaffarzadegan

**Affiliations:** 1 John Glenn College of Public Affairs, The Ohio State University, Columbus, Ohio, United States of America; 2 Grado Department of Industrial and Systems Engineering, Virginia Tech, Blacksburg, Virginia, United States of America; Iowa State University, UNITED STATES

## Abstract

While behavioral and social sciences occupations comprise one of the largest portions of the “STEM” workforce, most studies of diversity in STEM overlook this population, focusing instead on fields such as biomedical or physical sciences. This study evaluates major demographic trends and productivity in the behavioral and social sciences research (BSSR) workforce in the United States during the past decade. Our analysis shows that the demographic trends for different BSSR fields vary. In terms of gender balance, there is no single trend across all BSSR fields; rather, the problems are field-specific, and disciplines such as economics and political science continue to have more men than women. We also show that all BSSR fields suffer from a lack of racial and ethnic diversity. The BSSR workforce is, in fact, less representative of racial and ethnic minorities than are biomedical sciences or engineering. Moreover, in many BSSR subfields, minorities are less likely to receive funding. We point to various funding distribution patterns across different demographic groups of BSSR scientists, and discuss several policy implications.

## Introduction

As an occupational label, “behavioral and social sciences” includes a wide range of scientific fields that deal with how humans behave and make decisions in different social settings. In most coding schema, the label includes, but is not limited to, economics, political science, psychology, sociology, and anthropology. As do other professionals, the behavioral and social sciences research (BSSR) workforce contributes to the advancement of science in a wide range of strategic domains.

Past studies have stressed the role of behavioral and social sciences in the design and implementation of better public policies. Building trust and involving the community in the development and implementation of environmental policies, or the development of laws and regulations required for advancing controversial topics (such as stem cell research), are examples of “other sciences” benefiting from the social and behavioral sciences [1–3]. Cognitive biases and other psychological and decision making factors are known to be some of the major barriers in preventing environmental catastrophes such as global warming [4]. Moreover, the effectiveness of any science advisory committee depends on understanding and meeting the expectations of the public and overcoming political dynamics that may inhibit effective policy implementation, all being behavioral and social phenomena [1].

At present, a huge population of BSSR scientists is conducting research in the domains of health, education, domestic and international security, and technology-related areas such as green energy. Even scientists who study diversity in the scientific workforce are among the BSSR workforce population.

BSSR has made major contributions on the specific topic of health [5]. Behavioral and social scientists have studied patients’ willingness to receive treatment or comply with medical procedures, such as social-medical studies of mental illnesses in which factors such as friends and families’ behavior and social stigma are argued to influence medical treatments [6, 7]. Moreover, economic, management, or political science studies of health systems inform many health policy decisions (e.g., [8–10]), and these studies can contribute to higher quality of health services and social wellbeing. Effective treatments and public health advancements also come from the integration of biomedical and behavioral sciences. Thus, understanding the behavioral and social elements of health are critical to increase the nation’s health and is of interest to government agencies such as the U.S. National Institutes of Health [5].

Despite its importance, insufficient attention has been paid to the behavioral and social sciences workforce in comparison to other fields [11]. In a 2015 Executive Order, President Obama stressed the importance of applying insights from BSSR in policy making [12].

“By improving the effectiveness and efficiency of Government, behavioral science insights can support a range of national priorities, including helping workers to find better jobs; enabling Americans to lead longer, healthier lives; improving access to educational opportunities and support for success in school; and accelerating the transition to a low-carbon economy.” [12]

One implication of this lack of attention to BSSR is the absence of comprehensive quantitative studies of diversity in this population. Developing an effective strategic plan to maintain and improve the BSSR workforce requires an understanding of the major trends in the population, including racial/ethnic diversity and gender balance over time. Past studies discuss major trends in STEM fields with a focus on engineering, biomedical, or physical sciences [13–16]. Several of these studies point to concerns about the supply and demographic composition (gender or racial/ethnic imbalances) of the workforce in the engineering or biomedical sciences [13, 14, 17–20]. Another common concern is related to the productivity and demographic characteristics of young scholars and university students [21–25]. Whether the behavioral and social sciences suffer from the same imbalances as engineering, biomedical, or physical sciences is an empirical question. Moreover, it is critical to delve deeper into BSSR fields and ask whether the demographic trends apply to all of BSSR or only some specific fields within BSSR.

In this paper, we examine major trends in the population of BSSR scientists, funding distributions across different subfields of behavioral and social sciences, and the scientists’ productivity. Our study is descriptive, aimed at uncovering major racial/ethnic and gender-related patterns rather than identifying any causality. The goal is to evaluate the general trends and shed light on the differences between BSSR scientists and other scientists and engineers, as well as variations within BSSR in terms of population of researchers, diversity, and funding sources.

## Diversity in higher education

In this paper, diversity refers to demographic measures such as race/ethnicity (e.g., [26–28]) and gender (e.g., [27, 28]). Looking at the general U.S. population in 2013, the percentage of white non-Hispanic, Asian non-Hispanic, Black non-Hispanic, and Hispanic were 67 percent, 5 percent, 12 percent, and 14 percent, respectively [29]. The demographic distribution of scientists does not follow the same pattern. As might be expected, minorities and, in many fields, women are underrepresented in the population of scientists, which raises various concerns. In addition to the positive effects of diversity in academic performance [28], diversity by itself is considered a social value and an indicator of equal opportunity for different races and genders.

Several past studies have looked at diversity in the scientific workforce, mainly using general data from all fields or from fields other than the social sciences. A common finding is that we still do not have gender or racial/ethnic parity in the STEM workforce. Minorities are less likely to be promoted up the higher education ladder to full professor positions [30] or receive federal grants [20].

As for gender diversity, evidence points to the underrepresentation of women in math-intensive fields [31]. In geoscience, engineering, economics, mathematics, computer science, and physics, the dropout rates are higher for women than men both as students in graduate schools and as professionals during their careers [31]. This gender gap in the STEM workforce persists because of both employers’ behavior and employees’ personal preferences. Controlled experiments have shown that employers have gender biases against women when hiring for executive positions such as laboratory management, and women often receive lower salaries than their male counterparts [32]. Moreover, women in STEM are more likely to leave their occupations early on [33]. Career preferences, life choices [31], and stereotypes about career choices [34] are some of the main causes of the gender disparities in math-intensive fields. The fact that women are stereotypically expected to spend more time with their children [34] can explain gender imbalances.

Ceci et al. [18] compared different characteristics of men and women from early childhood through entry into the workforce and concluded that the gender gap has roots in pre-college training, when people’s career preferences are shaped. Women are argued to be more interested in organic or natural-oriented fields than other fields. Consequently, they are underrepresented in math-intensive fields compared to their strong representation in non-math-intensive fields. This underrepresentation persists at higher levels of education as well as in tenure-track faculty positions. Data suggest that women quit faculty jobs before tenure more so than men. The “leakage” is more significant in non-math-intensive fields, meaning that women are less interested in entering into math-intensive fields at the undergraduate level, but once they enter these fields they are more likely to persist in it and earn a doctorate degree [18].

Women are still underrepresented among faculty in math-intensive fields, comprising less than 20 percent of the faculty positions [18]. Even in the non-math-intensive fields, where women are better represented, a considerable percentage of women who earn a PhD do not land tenure-track positions. Although the current state of non-math-intensive fields is better in terms of gender diversity, the existing “pipeline leakages” in non-math-intensive fields reported by Ceci et al. show that these fields are still in need of further investigation and attention [18]. These studies also provide insights into the topic of gender diversity in BSSR. Some BSSR fields, especially economics, are considered to be math-intensive fields, and are thus expected to lack gender equity.

Similar reasons can also be offered for the lack of racial/ethnic parity in STEM fields [34]. Scholars and policy makers have increased their focus on the distribution of funding by different racial/ethnic groups–especially with recent academic work [20]. Ginther et al. [20] found an association between racial/ethnic demographics of NIH grant applicants and their chances of getting a proposal funded. Specifically, Ginther et al. [20] found that, controlling for various institutional factors, Asians are 4 percentage points and African-Americans are 13 percentage points less likely to be funded than whites. Ginther et al. [20] also found positive effects of prior NIH awards and journal citations on receiving NIH grants, which suggests a reinforcing loop of success for the already successful and a deteriorating trend regarding future chances for success of minorities [35]. As a result, NIH decided to assess carefully grant reviewers’ implicit bias against minorities [36].

Another common observation is the shortage of doctoral-degree holders from racial minorities in faculty positions. Gibbs et al. [37] compared representation of underrepresented minorities (URMs) in faculty positions with their representation in the pool of PhD graduates. They argue that the problem of diversity is in the transition, and while more minorities are receiving PhDs, many are not aspiring for faculty positions. Gibbs et al.’s data come from medical school basic science departments.

In one of the few studies of diversity in behavioral and social sciences, Boyle and colleagues [38] looked at funding in social sciences in the United Kingdom and found a relatively fair distribution of funding across male and female grant applicants (18% of female applicants and 18% of male applicants received funding). But they also note a self-filtering phenomenon: many female researchers do not apply for grants. This self-filtering means that women receive only 41 percent of total U.K. funding in the social sciences.

Since most past studies are about fields other than behavioral and social sciences, we know little about overall diversity among BSSR scientists. There is no reason to think that diversity in BSSR follows the same pattern as other STEM fields or that the topic is less important. As stated before, BSSR plays a critical role in societal wellbeing [5], and understanding BSSR population trends in terms of productivity and gender and racial/ethnic diversity is important. Furthermore, members of the BSSR workforce (such as political scientists and economists) take many high-level policy positions, and the lack of diversity can translate to a lack of representation for women and minorities in managerial positions.

Overall, the current studies show that diversity is still a major issue in the science workforce across some STEM fields. BSSR as a major STEM field is worthy of its own focus. Our intuition from reading past studies is that there are important differences in representation by gender or race within behavioral and social sciences subfields as well. A systematic analysis needs to uncover the current trends in the population of BSSR. The current study is a major step in this direction.

## Method

We use data from five Surveys of Doctorate Recipients (SDR) conducted by the U.S. National Science Foundation (NSF). It is a longitudinal survey designed to provide career history and demographic information about individuals with doctoral degrees from U.S. academic institutions who are U.S. residents at the time of the survey (i.e., U.S. citizens, permanent residents [green card holders], and temporary residents [visa holders]). The survey has been conducted since 1973. It includes individuals with doctoral degrees in the sciences, including social sciences, under age 76.

We use SDR data for surveys conducted in 2001, 2003, 2008, 2010, and 2013. Most of our analysis is based on the 2003 and 2013 data. The 2013 survey was selected as the most recent available data; the 2003 survey was selected as a baseline for comparison and examination of changes over a decade. Years 2001 and 2008 were the only years that questions about publications were included in the surveys, and are our sources of data for analyzing productivity measures.

The SDR includes such variables as date of birth, educational history, employment status, field of degree, geographic place of employment, occupation, labor force status, race/ethnicity, salary, sex, and many others. The SDR’s sample size is one of its strengths; the overall sample size for the 2013 survey was approximately 40,000, and just under 10 percent of survey respondents are selected for biennial interviews. Response rates are considered to be good; for example, 76 percent of those surveyed completed the survey in 2013. SDR survey weights, provided by NSF, are used in our analysis to adjust for attrition bias, resulting in more representative data and to estimate population measures. NSF has anonymized and de-identified the dataset to make it publicly available for research projects. The Ohio State University’s Behavioral and Social Sciences Institutional Review Board considers the analysis conducted here exempt.

To measure demographic characteristics, we use four major variables from this survey: gender, age, ethnicity, and citizenship status. We also use three outcome measures: the number of conference papers per person; the number of journal publications per person; and funding sources. For conference papers and journal publications, the survey asks respondents to report papers produced during the last five years. For funding sources, the survey asks respondents whether they had government funding during the past year and, if yes, what agency was the source of funding. It is reasonable to assume that a positive response to the funding question does not mean that the respondent was necessarily a “principal investigator” of a grant.

For our analysis of the BSSR workforce, we narrowed down the data to the fields of economics, sociology, psychology, political sciences, and other social sciences. Other major fields (biomedical, agricultural sciences, and engineering) are used for macro-level comparison. The detailed major fields of the dataset are as follows: psychology (educational psychology, clinical psychology, counseling psychology, experimental psychology, general psychology, and industrial and organizational psychology; social psychology; other psychology; other social sciences), economics (agricultural economics, economics), political sciences (public policy studies, international relations, political sciences and government), sociology, and other social sciences (area and ethnic studies, linguistics, anthropology and archeology, criminology, geography, history of science).

The analyses are all descriptive with the goal of uncovering major trends rather than specifying any causal factors.

## Demographics of the BSSR workforce

Tables 1 and 2 report major demographic variables for the entire group of BSSR scientists and different fields within BSSR for 2003 and 2013. We provide data on biological and agricultural sciences as well as engineering for comparison.

**Table 1:** Demographic trends in the BSSR workforce by gender, age, and citizenship.

**Major Fields**	**Population**		**Women**		**Average Age**		**U.S. Citizens**	
	2003	2013	2003	2013	2003	2013	2003	2013
**All BSSR**	**192,865**	**234,957**	**42%**	**48%**	**51**	**54**	**95%**	**94%**
Psychology	102,285	122,589	50%	56%	51	54	98%	98%
Economics	25,440	29,270	18%	24%	52	54	87%	83%
Political sci	20,523	25,812	26%	32%	52	54	95%	93%
Sociology	16,809	18,645	43%	52%	54	55	96%	96%
Other social sci	27,807	38,641	44%	49%	52	55	94%	93%
**Bio & agricultural sci**	**168,784**	**217,671**	**30%**	**41%**	**49**	**51**	**92%**	**90%**
**Engineering**	**117,204**	**155,404**	**9%**	**16%**	**49**	**49**	**83%**	**78%**

Note: SDR sample sizes for years 2003 and 2013, respectively, are: All BSSR: 8,217 and 8,822; Psychology: 4,136 and 4,454; Economics: 1,048 and 1,081; Political science: 863 and 951; Sociology: 871 and 808; Other social sciences: 1,299 and 1,528; Bio & agricultural sciences: 7,570 and 8,060; Engineering: 4,817 and 5,410.

**Table 2:** Demographic trends in the BSSR workforce by race.

Major Fields		White		Asian		Black		Hispanic		Other	
		2003	2013	2003	2013	2003	2013	2003	2013	2003	2013
**All BSSR**		**86%**	**82%**	5%	7%	4%	5%	3%	4%	2%	2%
** **	Psychology	89%	85%	2%	4%	4%	5%	3%	5%	2%	2%
** **	Economics	79%	74%	14%	17%	3%	4%	3%	4%	1%	1%
** **	Political sci	85%	82%	5%	7%	6%	6%	2%	3%	2%	2%
** **	Sociology	84%	81%	6%	6%	6%	7%	3%	4%	1%	2%
** **	Other social sci	82%	80%	7%	7%	4%	4%	3%	5%	3%	3%
**Bio & agricultural sci**		**80%**	**75%**	**14%**	**17%**	**2%**	**3%**	**3%**	**4%**	**1%**	**2%**
**Engineering**		**64%**	**56%**	**31%**	**38%**	**2%**	**2%**	**2%**	**3%**	**1%**	**1%**

As Table 1 shows, more than half of the within BSSR workforce members are psychology graduates. In the past decade, the number of PhD graduates in BSSR overall and within psychology have both increased by about 20 percent. Other fields have also experienced growth, with economics being the largest BSSR field after psychology, comprising about 12 percent of the total.

Tables 1 and 2 also show changes in diversity-related measures for different fields. In all BSSR fields, the percentage of women PhD graduates has increased from 42 to 48 percent. There is more gender balance in behavioral and social sciences than in biomedical or engineering fields. However, there is considerable variation within subfields of behavioral and social sciences. In psychology and sociology, most PhD graduates are now women (56% and 52%, respectively), whereas the percentages in economics and political science are comparatively low (24% and 32%, respectively).

Table 1 also presents data on the average age and citizenship of scientists. The average age of BSSR scientists is three years higher than biomedical scientists and five years higher than engineering PhDs. We also analyzed the age distribution of scientists (see S1 Appendix). In short, about one third of BSSR scientists are over 60 years old; the average age of women in BSSR is much lower than men; and the average age of URMs in BSSR is much less than whites and Asians.

Furthermore, Table 1 reports that a strong majority of BSSR PhD holders are U.S. citizens–even more than in biomedical sciences and engineering. The main outlier in BSSR is economics, with about 17 percent non-US citizens.

There is less racial diversity in BSSR than in engineering and biological sciences (Table 2). Among all BSSR workers, the percentage classified as non-whites has increased since 2003. In 2013, 18 percent of the BSSR workforce were non-white. This represents a 4-percentage-point growth between 2003 and 2013. The most diverse field is economics, in which 26 percent of PhD holders are non-white. Psychology as a discipline has the lowest percentage of minorities. One important observation is that Asians comprise a relatively small number of behavioral and social sciences workers, as opposed to the biomedical and engineering fields. If we focus on URMs (non-whites excluding Asians), we note their lack of representation in all behavioral and social sciences, including economics.

We also looked at the same demographic measures among incoming PhDs, defined as individuals who got their PhD degrees within four years of the time of the survey. Tables 3 and 4, which report the results, show that among new PhDs, psychologists and sociologists are mostly women (70% and 60%, respectively). If this trend continues, we will see gender disparities (significantly more women than men) among the total population of scientists working in those fields. Table 3 also shows that political science has achieved gender balance among new graduates, while economics is still dominated by men. In comparison to biomedical and engineering STEM fields, we see more women among new PhD graduates in behavioral and social sciences.

**Table 3:** Demographic trends of incoming PhDs in BSSR workforce by gender, age, and citizenship.

Major Fields		Women		Average Age		U.S. Citizens	
		2003	2013	2003	2013	2003	2013
**All BSSR**		**58%**	**59%**	**38**	**36**	**88%**	**86%**
** **	Psychology	70%	70%	38	35	95%	93%
** **	Economics	33%	36%	35	35	61%	62%
** **	Political sci	36%	46%	39	38	91%	79%
** **	Sociology	65%	60%	39	36	85%	94%
** **	Other social sci	51%	57%	40	38	84%	87%
**Bio & agricultural sci**		**45%**	**52%**	**35**	**34**	**75%**	**75%**
**Engineering**		**18%**	**23%**	**35**	**34**	**49%**	**49%**

**Table 4:** Demographic trends of incoming PhDs in BSSR workforce by race.

Major Fields		White		Asian		Black		Hispanic		Other	
		2003	2013	2003	2013	2003	2013	2003	2013	2003	2013
**All BSSR**		**76%**	**69%**	**10%**	**14%**	**7%**	**5%**	**5%**	**8%**	**3%**	**2%**
** **	Psychology	79%	74%	7%	8%	7%	6%	5%	9%	2%	3%
** **	Economics	58%	55%	29%	30%	7%	4%	6%	10%	0%	1%
** **	Political sci	76%	67%	6%	17%	11%	10%	4%	5%	2%	1%
** **	Sociology	73%	68%	10%	14%	11%	10%	5%	5%	0%	3%
** **	Other social sci	80%	70%	10%	12%	3%	5%	4%	9%	3%	3%
**Bio & agricultural sci**		**67%**	**61%**	**24%**	**26%**	**4%**	**5%**	**4%**	**6%**	**1%**	**2%**
**Engineering**		**49%**	**43%**	**44%**	**47%**	**3%**	**2%**	**3%**	**5%**	**0%**	**3%**

Note: race categories are not limited to U.S. citizens; for example, the category “white” includes U.S. citizens and non-citizens.

Racial/ethnic disparities still persist among new PhD graduates. Fewer blacks work in most subfields, especially in psychology and economics. Political science and sociology are diversifying over time, but there is a long way to go before we see racial/ethnic parity.

In sum, it seems that the demographic patterns among BSSR scientists are different than the same measures for biomedical scientist and engineering PhD holders. In BSSR, we observe more racial/ethnic disparities than other fields. In contrast, BSSR exhibits greater gender balance within fields, as opposed to biomedical sciences and engineering. Furthermore, there is great variation in demographic composition across different subfields for BSSR workers.

## Productivity and funding sources for different demographic groups

We examined the types of research funding and productivity outcomes among different racial/ethnic groups and genders within the BSSR workforce. In this section, we first provide descriptive statistics and then conduct a series of statistical analyses to investigate associations between demographic variables and the likelihood of recent funding and recent publications. We narrow the analysis to those of BSSR scientists who are employed in tenure-track and tenured positions in academia. There are, of course, fewer incentives for researchers outside of academia than for those inside academia to publish papers or apply for research grants. Our analysis of funding distribution and productivity among all BSSR workforce (see S2 Appendix) supports the intuition that having an academic position is a significant predictor of receiving funding, having a paper published, and giving a conference presentation.

As Table 5 reports, the BSSR workforce has benefited considerably from government funding. The column “Any Government Funding” reports the percentage of doctorate holders supported by a U.S. government grant in 2003 and 2013 and shows a slight drop over that span of time. As stated, the data are not specifically about principal investigators, and include all other potential roles such as co-investigators. Among the subfields, psychologists benefit the most; about one-third received government funding. Furthermore, about one-fifth of economists and sociologists received government funding. The lowest percentage was for political scientists.

**Table 5:** Funding and productivity measures for BSSR.

Major Fields	Funding										Productivity			
	Any Gov. Funding		NIH		NSF		Department of Education		Department of Defense		Average Last Five Years Publication		Average Last Five Years Conference Papers	
	2003	2013	2003	2013	2003	2013	2003	2013	2003	2013	2001	2008	2001	2008
**All BSS**	**26%**	**24%**	**10.5%**	**10.4%**	**5.3%**	**6.7%**	**2.8%**	**2.8%**	**1.4%**	**1.7%**	**5.5**	**6.5**	**9.5**	**10.5**
Psychology	34%	33%	22.3%	21.2%	4.8%	6.1%	2.7%	4.1%	2.7%	2.4%	7.5	9.0	12.0	13.0
Economics	25%	21%	2.4%	5.2%	7.7%	7.9%	1.8%	2.0%	1.0%	0.9%	5.5	5.0	8.0	8.0
Political sci	11%	12%	1.3%	1.6%	2.9%	1.9%	3.0%	1.9%	0.5%	1.9%	3.5	4.0	7.0	8.0
Sociology	22%	20%	7.9%	6.6%	3.6%	7.6%	3.5%	2.3%	0.5%	1.2%	4.5	6.0	8.5	10.0
Other social sci	26%	22%	4.1%	4.9%	7.6%	9.8%	3.4%	2.1%	0.7%	1.0%	5.0	5.5	9.5	9.5

Note: Some government funding sources are excluded from this table due to the small size of their contributions. In some cases, people receive government funding from multiple sources or from state governments.

Table 5 also reports data from four specific U.S. federal agencies that are among the major sources of U.S. government research spending. As the table shows, the National Institutes of Health (NIH) provides a considerable portion of government funding and supports about one-tenth of the BSSR academic workforce. The percentage of BSSR scientists who have been supported by NIH during the past decade has been relatively constant. However, most NIH funding goes to psychologists. In 2013, about one-fifth of psychologists received NIH funding. Other subfields benefit to a significantly lesser degree: about 5–7 percent of economists, sociologists, and “other social scientists” receive NIH funding, and for political scientists the rate is only 1.6 percent.

NSF grants, one of the main sources of funding for BSSR fields, supported about 6.7 percent of the BSSR workforce in 2013. NSF funding is awarded to a smaller portion of political scientists (1.9%) than psychologists (6.1%), economists (7.9%) and sociologists (7.6%). It is also notable that the proportion of all those in BSSR fields receiving NSF funding has increased, except for political science.

The table also reports data for funding by the U.S. Departments of Education (DoE) and Defense (DoD). The DoE funded 2.8 percent of BSSR scientists in 2013. As might be expected, psychologists receive the most funding (4.1%). Funding distribution from DoD is relatively small and around 1–2 percent across different BSSR fields. The Department of Energy (not shown in the table) funds a very small portion of BSSR scientists, less than 1 percent.

Productivity in Table 5 is measured by publications and conference papers. Questions about the number of publication and conference papers (specifically, about publications and conference papers during the past five years) were asked only in the 2001, 2003, and 2008 surveys. Overall, there is a slight increase in publication and conference papers in most subfields. Among all BSSR academic staff, the average annual number of last five years’ conference papers per person increased from 9.5 in 2001 to 10.5 in 2008. During the same period, the average annual number of last five years’ publications per person also grew, from 5.5 publications to 6.5. We see more variation between different subfields.

**Fig 1:**
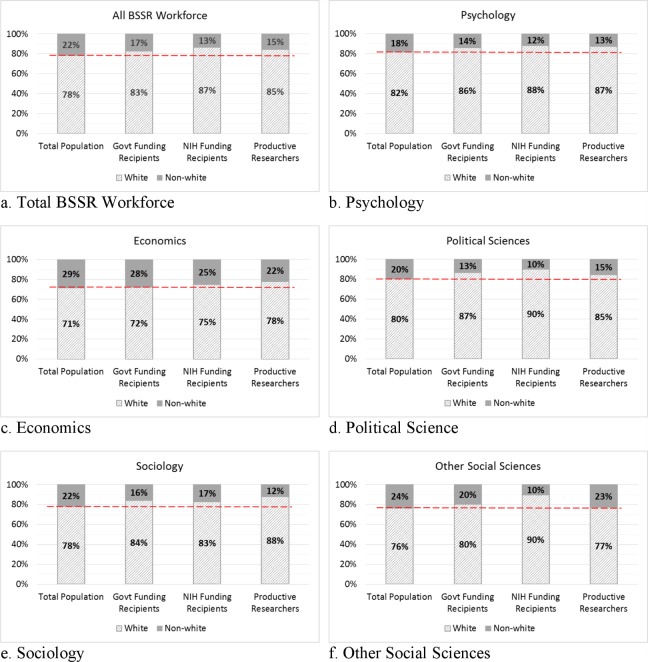
Racial/ethnic distribution of scientists in academic positions by major field. Source: SDR 2008 and 2013.

Additionally, we analyze gender and racial distribution among funding recipients and productive researchers. Fig 1 compares white vs. non-white researchers among the entire group of BSSR in academia (1a) and for the subfields within BSSR (1b-1f) in terms of funding distribution and productivity. In each graph, moving from left to right, the bars show: the distribution of the population (percentage white vs. non-white); the distribution of the population that received government funding; the distribution of people who received NIH funding; and the distribution of people who wrote more papers than the average number of papers in their field. We label the last group as “productive researchers.” We would expect the distributions depicted in the second, third, and fourth bars to be close to the first bar, as the dashed lines indicate. For example, Fig 1A shows that 78 percent of BSSR scientists in academia are white; if we look only at those who received government funding, 83 percent are white.

Overall, Fig 1 shows the dominance of whites in terms of funding and publications. In all fields, a significant portion of the majority is white and the rate at which funding is distributed favors the majority even more. Similar discrepancies exist for productivity. For example, as Fig 1a shows, 85 percent of the population of relatively productive BSSR scientists are white.

**Fig 2:**
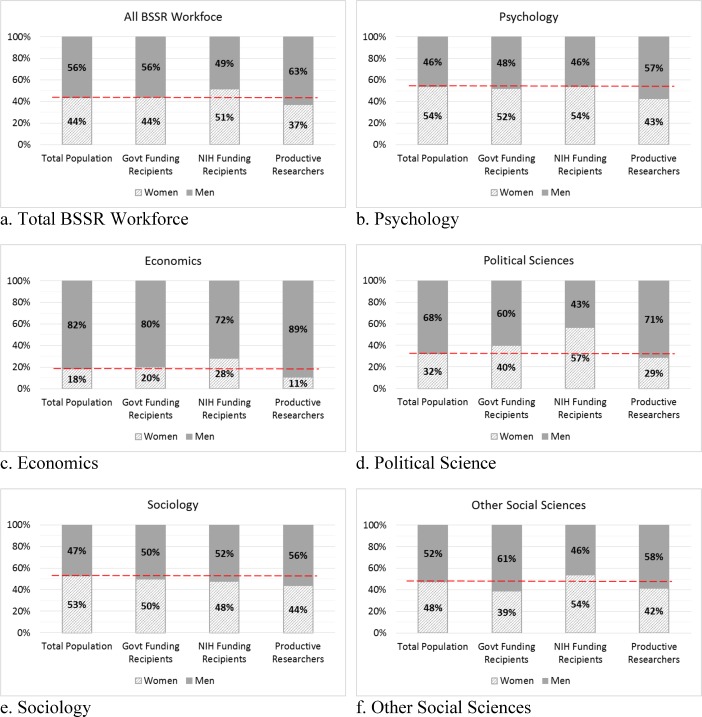
Gender distribution by major field. Source: SDR 2008 and 2013.

Fig 2 shows the gender balance in terms of funding distribution and productivity and, as in the previous figure, calculates the values for funding and productivity. Fig 2A shows that there is an overall gender balance in the BSSR workforce. The balance is also observable among the subsample of BSSR researchers receiving government funding: 44 percent of BSSR professors are women and 44 percent of funding recipients are women. Notably, 51 percent of NIH grant recipients in BSSR fields are women. Among productive researchers (those who wrote more papers than the average in their fields), 37 percent are women.

There are substantial variations among subfields of BSSR in the gender and racial/ethnic composition of scientists that receive funding (Fig 2B–2F). While men constitute the majority of the workforce in economics and political science, women receive a larger proportion of both government and NIH funding. In political science, 32 percent of professors are women, while 40 percent of government funding recipients are women and 57 percent of NIH grant recipients are women. It is very encouraging that the gender gaps in funding for BSSR scientists are potentially smaller, and this might also relate to more women doing research in areas that are more fundable by NIH or NSF. Determining the cases of this apparent parity in funding for BSSR scientists deserves more direct study using data sources that allow much more fine-grained investigation of individual research funding.

There remain gaps in reported research productivity at the field level, although among BSSR scientists overall the differences between women and men in terms of publications are relatively small. For example, the fields of psychology and sociology are 54 and 53 percent women, respectively, while the percentages of women scientists among productive scientists in those fields declines to 43 and 44 percent, respectively.

In summary, the gaps among scientists in terms of funding by gender have narrowed, but there remains an apparent productivity gap for scientists by gender. Women may be receiving the same or greater funding than men in the same fields, but there are gaps in publishing. However, we observe that URMs are disproportionally receiving less funding, a pattern that warrants further policy considerations. Examining potential causal factors that create these gaps is an important research problem and will require substantially more detailed data.

Finally, we investigate more systematically funding distribution and productivity measures by racial/ethnic group and gender in BSSR–while still considering potential differences by subfield. The focus remains on BSSR scientists employed in tenure-track and tenured positions in academia. Again, S2 Appendix shows that people with academic positions receive more funding, write more papers, and go to more conferences.

We conduct a logistic regression to estimate the likelihood of receiving a federal grant (Table 6) and report odds ratios. The only difference in the models is the sample of analysis; the first model looks at all BSSR, while the others look at each subfield. In all models, the main independent variables are Female, Asian, and URMs. We control for academic rank (assistant, associate, or full professor) as well as for some other variables not shown in the table (listed in the table note). In the first model, we also include controls for subfields with dummy variables. The results for the main variables are shown in Table 6; (full results are reported in S3 Appendix).

**Table 6:** Associations between demographic variables and the likelihood of receiving government funding among BSSR scientists employed in tenure-track or tenured positions.

	Likelihood of receiving government funding				
VARIABLES	All BSSR	Psychology	Economics	Political Sci	Sociology
Female	0.85	0.88	1.13	1.13	0.83
	(0.09)	(0.14)	(0.37)	(0.40)	(0.24)
Race: (Ref: White)					
Asian	0.55***	0.79	0.54	0.58	0.26**
	(0.10)	(0.23)	(0.21)	(0.31)	(0.14)
URM	0.61***	0.49***	1.31	0.23***	0.55
	(0.08)	(0.10)	(0.43)	(0.13)	(0.21)
Profs: (Ref: Assistant Prof.)					
Associate Prof.	0.82	0.78	1.01	1.31	0.60
	(0.11)	(0.16)	(0.40)	(0.56)	(0.25)
Professor	1.06	0.99	2.16*	0.58	0.66
	(0.17)	(0.24)	(0.90)	(0.32)	(0.33)
Constant	0.29*	0.14*	0.28	0.17	1.85
	(0.18)	(0.14)	(0.41)	(0.20)	(2.21)
Pseudo R^2^	0.09	0.07	0.13	0.08	0.08
Observations	2,841	882	538	433	364

*** p<0.01

** p<0.05

* p<0.1

Notes: The table reports odds ratios. Standard errors are in parentheses. The models are logistic regressions, and the dependent variable is AnyGovFunding, which is equal to 1 if the individual received any federal funding during the past year and otherwise is equal to 0. Data are SDR 2013. Other variables controlled for in the regressions are Citizenship (US vs. Non-US), Age, Marriage, Children, Spousework (whether or not spouse is employed), Work Duration, and Employer Size. We controlled for across subfield variations in all models with dummy variables for each subfield. The complete results are reported in Table A2 in S3 Appendix.

None of the models show any gender effect, but do some show racial disparities. As the first model shows, Asians and URMs are less likely to receive government funding. The size of the effect is considerable; the chances of Asians and URMs receiving funding are about 55 and 61 percent, respectively, of whites. The next models show the patterns for each subfield. In psychology and in political science in particular, URMs are less likely to receive funding than whites; in sociology, Asians are less likely to receive funding than whites. These points resonate with our observations from descriptive statistics: funding is fairly distributed across different genders, but URMs and Asians receive less funding than whites, at least in some of the subfields of behavioral and social sciences.

We conduct a similar analysis for publication (Table 7) and conference papers (Table 8). The models are linear regressions. Similarly, in all models, the main independent variables are Female, Asian and URM. We control for academic rank. We also control for more variables not shown in the table (listed in the table note). In the first model, we also include controls for subfields with dummy variables. (Full results are reported in S4 Appendix.)

**Table 7:** Associations between demographic variables and publications among BSSR scientists employed in tenure-track or tenured positions.

	Journal publications				
VARIABLES	All BSSR	Psychology	Economics	Political Sci	Sociology
Female	-1.06***	-1.09	-1.53*	0.52	-1.97**
	(0.39)	(0.89)	(0.82)	(0.64)	(0.92)
Race: (Ref: White)					
Asian	-1.04	-1.67	-1.93*	-0.52	-1.19
	(0.67)	(1.78)	(0.99)	(1.20)	(1.77)
URM	-1.60***	-1.62	-1.38	-1.17	-2.48**
	(0.46)	(1.13)	(0.98)	(0.73)	(1.06)
Profs: (Ref: Assistant Prof.)					
Associate Prof.	2.40***	3.10***	3.93***	1.01	2.21*
	(0.50)	(1.16)	(1.02)	(0.77)	(1.23)
Professor	5.26***	7.25***	6.27***	3.23***	4.87***
	(0.59)	(1.40)	(1.15)	(0.89)	(1.46)
Constant	7.74***	6.50	10.70**	2.11	-0.02
	(2.58)	(5.72)	(4.84)	(4.36)	(6.92)
R^2^	0.10	0.06	0.10	0.04	0.11
Observations	2,296	769	364	385	329

*** p<0.01

** p<0.05

* p<0.1

**Table 8:** Associations between demographic variables and conference papers among BSSR scientists employed in tenure-track or tenured positions.

	Conference papers				
VARIABLES	All BSSR	Psychology	Economics	Political Sci	Sociology
Female	-0.48	0.24	-0.59	0.50	-3.29**
	(0.53)	(1.11)	(1.31)	(1.10)	(1.46)
Race: (Ref: White)					
Asian	-2.10**	-2.07	-3.17**	-1.29	-4.39
	(0.92)	(2.23)	(1.58)	(2.07)	(2.81)
URM	-2.31***	-3.64**	-0.66	-2.94**	-3.61**
	(0.63)	(1.41)	(1.57)	(1.26)	(1.68)
Profs: (Ref: Assistant Prof.)					
Associate Prof.	2.63***	4.53***	1.43	0.71	2.91
	(0.69)	(1.45)	(1.63)	(1.33)	(1.95)
Professor	6.02***	9.29***	6.03***	3.28**	6.00**
	(0.82)	(1.76)	(1.83)	(1.54)	(2.33)
Constant	17.70***	25.95***	13.88*	12.67*	12.06
	(3.57)	(7.17)	(7.73)	(7.53)	(10.99)
R^2^	0.07	0.07	0.04	0.05	0.05
Observations	2,296	769	364	385	329

*** p<0.01

** p<0.05

* p<0.1

Notes: Standard errors are in parentheses. The models in Tables 7 and 8 are linear regression models. The dependent variables are number of publications and number of conference papers during the past five years. Data from SDR 2008 (the latest survey that asked publication and conference paper questions); only individuals employed in tenure-track or tenured positions in academia. Other variables controlled for in the regressions are Citizenship (U.S. vs. Non-U.S.), Age, Marriage, Children, Spouse’s work (whether spouse is employed), Work Duration, and Employer Size. The complete results are reported in Tables A3 and A4 in S4 Appendix.

Academic rank is associated with more journal publications and conference papers. The first model shows that women publish about 1 less paper over five years and minorities publish about 1.6 fewer papers than whites over five years. In the subfields economics and sociology, women publish about 1.5 and 2 fewer papers, respectively, than men over five years. In terms of publications, there is no significant difference between URMs and whites in any of the subfields other than sociology.

Table 8 shows disparities in terms of conference papers. Asians and URMs have 2.1 and 2.3 fewer conference papers, respectively, in comparison to whites over a five-year period. In the fields of psychology, political sciences, and sociology in particular, URMs have fewer conference papers than whites. Lower representation in conferences can lead to fewer grants and fewer published journal papers.

In sum, it seems that a common pattern across all BSSR subfields is that there is no significant bias against women in term of funding. However, in some fields, minorities are less likely to get funding and have fewer publications or conference papers.

## Discussion and conclusion

This study contributes to the literature of science policy and labor economics by examining recent trends in the behavioral and social sciences workforce. Behavioral and social sciences are considered science fields, although when science policy scholars talk about the problems with the STEM workforce and education their data often come from physical or biomedical sciences and their attention is focused on non-BSSR scientists. Maintaining a strong and diverse workforce in BSSR, however, is required for nations to achieve their strategic plans for science, and thus more attention is required for better analyzing and evaluating the status of the BSSR workforce. We took a step in this direction by focusing on the U.S. BSSR workforce and analyzing population level trends and diversity-related heterogeneities among its scientists. We show that most population measures of the BSSR workforce are different than for other STEM fields.

We built on work from other studies on the problems of gender or racial/ethnic diversity in the science workforce [18] by examining productivity measures specifically for behavioral and social scientists. The BSSR workforce does suffer from various diversity-related challenges and issues related to funding shortages or funding distribution. Moreover, the average age of the BSSR workforce has been increasing, which may be due to BSSR scientists postponing retirement or taking longer to obtain their PhDs and secure academic positions [39]. In terms of productivity, we observe a growth pattern in almost all fields within BSSR. Our analysis shows that the average annual numbers of conference papers and journal publications have been increasing.

There are different indicators with regards to funding patterns. In 2013, about one-fourth of BSSR professors received some sort of government funding: About 10 percent of the population is supported by NIH and about 7 percent by NSF. Overall, a slightly lower proportion of all BSSR scientists receive government funding than was the case ten years ago. Furthermore, the pattern of funding distribution is different for different racial/ethnic groups. Psychologists are the most likely and political scientists are the least likely to receive government funding.

One important finding of this study is that while the BSSR workforce has diversity-related problems, their nature is different than those found in other science and engineering fields. Racial diversity is lower in all BSSR fields, and it appears that some subfields are predominantly white. The magnitude of this issue varies slightly across different fields. This matter becomes even more important if one considers that social and behavioral scientists in different fields all study human behavior and their interactions, and all can speak to the topic of equality and fairness through different paradigms. A fair racial and gender representation of the general public in the population of BSSR scientists would ensure better understanding of the issues underrepresented communities may face.

We observe relatively even gender spread in the full BSSR population, but at the level of subfields we see considerable heterogeneities and imbalances. Economics and political science are male-dominated fields. The population of incoming new PhDs is more gender-balanced, but it will take time to see a balance in the entire population of these subfields. We showed that economics has significantly fewer female PhDs than other fields, which resonates with other sources that argue more gender imbalance in math-intensive fields [18]. We also observe that female BSSR scientists are less productive across most fields. A future study should investigate further the gender-productivity issue in BSSR.

Finding policy tools to strengthen the BSSR workforce is not an easy task. Education is a complex system [40] and creating programs and processes that may help increase diversity is a challenge in higher education [25]. A primary tool the government can use is to provide increased research funding through NSF, NIH, or similar agencies, targeting specific demographics of students or researchers [41]. As previous studies have shown, aggregate levels of funding are blunt policy tools and their effect on the scientific workforce is questionable [41–43]. For example, in the biomedical sciences, many universities expanded their research capacities by investing in large buildings and laboratories in an effort to secure more funding. This approach proved ineffective, since sustaining growth resulted in the need for even more funding [44].

Any change in funding policy that responds to our major finding of diversity-related problems requires an evaluation of which portion of the population of BSSR scientists will actually receive the funding. This is an important concern, since “success for the successful” implies that the stream of funding is more likely to go to groups that have traditionally secured more funding, thereby exacerbating the diversity problem. Targeted funding for young scholars and minorities can help in this regard. Enterprise-level policies such as targeted mentoring projects are also crucial for the long-term success of small groups [45]. However, replicating diversity-related policies designed for other science and engineering fields may be a mistake since BSSR diversity problems are unique. Psychology and sociology have more problems of racial/ethnic diversity problems, whereas engineering fields are more plagued by gender-diversity issues. The BSSR fields of economics and political science lack both gender and racial/ethnic diversity.

This study provides first-order insights and a view of the big picture relating to major trends in the BSSR workforce, with a focus on diversity-related patterns. We recognize several limitations. Data are sources of limitation. In our case, it is difficult to track each individual researcher over time. One significant area that warrants further analysis is the measurement of productivity amongst BSSR scientists. We believe that, ideally, multiple measures are needed to evaluate scientific workforce performance, productivity, and diversity. While we discussed the patterns of publication among different groups of researchers, we believe that the quality of research is a much more complex outcome to consider. One needs to evaluate performance based on multiple measures of impact, especially considering the long delays that may occur before a scientific discovery is most effective. Furthermore, diversity is a multi-dimensional concept that goes beyond gender and racial or ethnic diversity. We simplified this categorization to offer a first-order insight. Future studies that include more detailed analysis and differentiate between different minority groups, age groups, and country of origin will be beneficial. Future studies can benefit from further analyzing different subfields of BSSR. For example, the trends in clinical psychology may be different than those in educational psychology, and both may differ from practice-oriented subfields that grant the doctor of psychology (PsyD) degree rather than a PhD. Our goal, here, was to offer a broader view, illuminating several indicators that require the careful consideration of researchers and policymakers.

## Supporting information

S1 Appendix Age distribution. A1 and A2 Figs in this appendix show the age distribution of BSSR workforce in comparison to biomedical scientists and engineering PhDs.
(DOCX)

S2 Appendix Association between having an academic position and productivity measures. A1 Table depicts results of logistic regressions for estimating the chance of receiving any government funding, having a publication, and a conference paper among all BSSR scientists (not just the ones in academic positions).
(DOCX)

S3 Appendix Complete Version of Table 6. A2 Table shows full results of Table 6.
(DOCX)

S4 Appendix Complete Version of Tables 7 and 8. A3 and A4 Tables show full results of Tables 7 and 8.
(DOCX)
