# Supplementary material for: Recent trends in the U.S. Behavioral and Social Sciences Research (BSSR) workforce
Source: PLoS One. 2017 Feb 6;12(2):e0170887. doi: 10.1371/journal.pone.0170887 (PMC5293198; doi:10.1371/journal.pone.0170887)
Supplement: S1 Appendix — A1 and A2 Figs in this appendix show the age distribution of BSSR workforce in comparison to biomedical scientists and engineering PhDs. (DOCX) [file pone.0170887.s001.docx]

**S1 Appendix. Age distribution.**

A1 Fig shows age histograms of BSSR workforce and compares them with biomedical (A1a Fig) and engineering (1Ab Fig). BSSR workforce is relatively an older workforce compared to biomedical sciences and engineering.

| 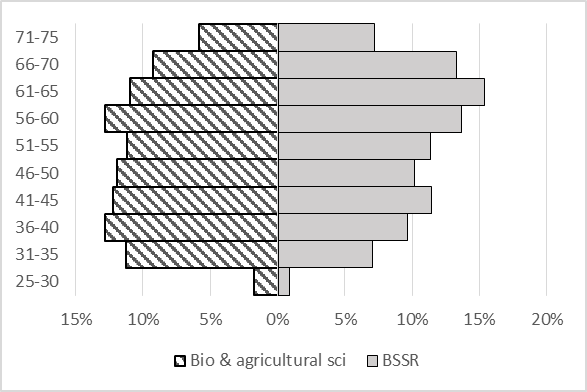 | 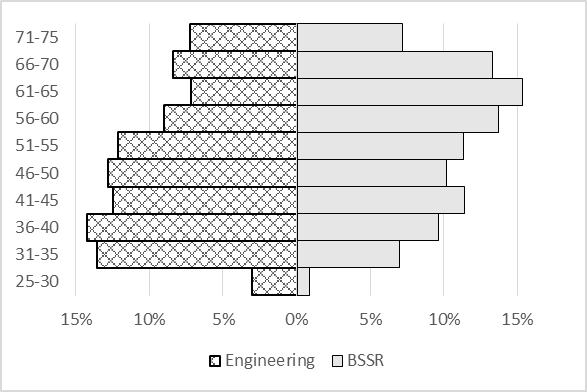 |
| --- | --- |
| (a) | (b) |

**Fig A1. Age distribution of BSSR scientists in comparison to biological and agricultural sciences (a) and engineering PhD workforce (b).**

A2 Fig reports age distribution of BSSR workforce by gender and racial groups (A2a and A2b Figs, respectively). Women have lower average age and a more uniform distribution than other groups.

| 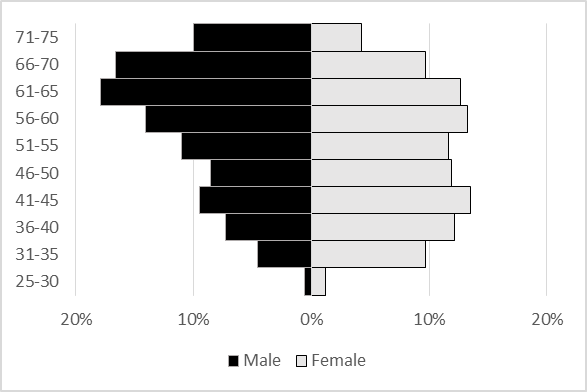 | 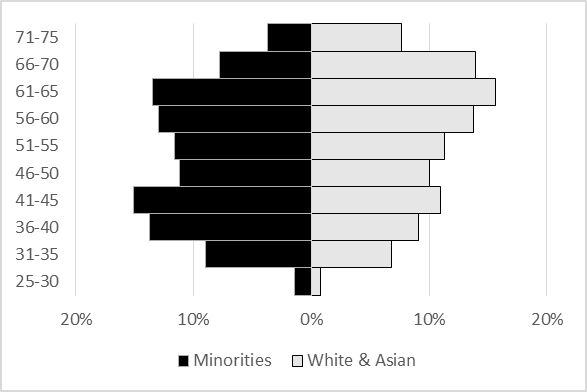 |
| --- | --- |
| (a) | (b) |

**Fig A2. Age distribution of BSSR scientists for different demographics.**
