# Supplementary material for: Recent trends in the U.S. Behavioral and Social Sciences Research (BSSR) workforce
Source: PLoS One. 2017 Feb 6;12(2):e0170887. doi: 10.1371/journal.pone.0170887 (PMC5293198; doi:10.1371/journal.pone.0170887)
Supplement: S2 Appendix — A1 Table depicts results of logistic regressions for estimating the chance of receiving any government funding, having a publication, and a conference paper among all BSSR scientists (not just the ones in academic positions). (DOCX) [file pone.0170887.s002.docx]

**S2 Appendix. Association between having an academic position and productivity measures.**

In A1 Table, we present an analysis for the likelihood of funding, having a publication, and a conference paper by including all individuals, whether or not they are employed in academia (M1-M3) and two more linear regressions for number of publications and number of conference papers (M4-M5). This provides a face validity that employment in academia is significantly associated with more funding, publications, and conference papers. In the paper (Tables 4 and 5) the analysis is among BSSR scientists who are employed in tenure-track or tenured positions in academia.

**Table A1. Associations between demographic variables and funding, publications, and conference papers among all BSSR scientist, whether or not employed in academic positions.**

|  | M1 | M2 | M3 | M4 | M5 |
| --- | --- | --- | --- | --- | --- |
| VARIABLES | Fund | Publication | Conference | Publication | Conference |
| Female | -0.0851 | -0.353*** | -0.329*** | -0.0663*** | -0.0551*** |
|  | (0.0686) | (0.0662) | (0.0695) | (0.0127) | (0.0121) |
| Race: (Ref: White) |  |  |  |  |  |
| Asian | -0.436*** | -0.126 | -0.150 | -0.0243 | -0.0236 |
|  | (0.120) | (0.117) | (0.128) | (0.0227) | (0.0217) |
| URM | -0.238*** | -0.327*** | -0.0974 | -0.0631*** | -0.0157 |
|  | (0.0852) | (0.0827) | (0.0888) | (0.0161) | (0.0153) |
| **Academic** | **0.233***** | **1.204***** | **1.406***** | **0.265***** | **0.273***** |
|  | **(0.0799)** | **(0.0733)** | **(0.0778)** | **(0.0149)** | **(0.0142)** |
| Citizenship: US | 0.804*** | -0.191 | -0.563*** | -0.0331 | -0.0623** |
|  | (0.126) | (0.147) | (0.183) | (0.0271) | (0.0259) |
| Major: (Ref: Psychology) |  |  |  |  |  |
| Economics | -0.244** | 0.565*** | 0.590*** | 0.117*** | 0.112*** |
|  | (0.106) | (0.0992) | (0.107) | (0.0193) | (0.0184) |
| Political science | -0.619*** | 0.190* | 0.397*** | 0.0412** | 0.0766*** |
|  | (0.123) | (0.102) | (0.111) | (0.0200) | (0.0191) |
| Sociology | -0.182 | 0.547*** | 0.733*** | 0.109*** | 0.127*** |
|  | (0.119) | (0.115) | (0.130) | (0.0219) | (0.0209) |
| other social sciences | -0.0652 | 0.517*** | 0.681*** | 0.104*** | 0.123*** |
|  | (0.0904) | (0.0923) | (0.102) | (0.0179) | (0.0171) |
| Age | -0.000126 | -0.0498*** | -0.0520*** | -0.00984*** | -0.00915*** |
|  | (0.00376) | (0.00380) | (0.00402) | (0.000722) | (0.000688) |
| Marriage | 0.0229 | 0.0344 | -0.00107 | 0.00554 | 0.000204 |
|  | (0.122) | (0.121) | (0.129) | (0.0233) | (0.0222) |
| Children | 0.00657 | -0.0933 | -0.184** | -0.0183 | -0.0293** |
|  | (0.0707) | (0.0672) | (0.0714) | (0.0130) | (0.0124) |
| Spousework | 0.193** | 0.0576 | -0.0703 | 0.0112 | -0.00900 |
|  | (0.0818) | (0.0782) | (0.0819) | (0.0151) | (0.0143) |
| Work Duration | -0.000790** | 0.000832*** | 0.000837*** | 0.000177*** | 0.000165*** |
|  | (0.000338) | (0.000307) | (0.000315) | (6.01e-05) | (5.73e-05) |
| Employer Size: (Ref: 1-99) |  |  |  |  |  |
| 100-4999 | 0.319*** | 0.323*** | 0.214** | 0.0706*** | 0.0773*** |
|  | (0.114) | (0.0990) | (0.0965) | (0.0194) | (0.0185) |
| 5000+ | 0.627*** | 0.851*** | 0.601*** | 0.180*** | 0.144*** |
|  | (0.105) | (0.0920) | (0.0898) | (0.0183) | (0.0174) |
| Constant | -2.525*** | 1.561*** | 2.754*** | 0.781*** | 0.905*** |
|  | (0.270) | (0.272) | (0.307) | (0.0519) | (0.0494) |
| Pseudo R^2^ | 0.0349 | 0.1739 | 0.1906 | 0.2202 | 0.2278 |
| Observations | 6,502 | 5,553 | 5,553 | 5,553 | 5,553 |

*** p<0.01, ** p<0.05, * p<0.1

Notes: Coefficients are reported (not odds ratios). Standard errors in parentheses. Dependent variable for M1 is AnyGovFunding which is equal to 1 if the individual received any federal funding during the past year, otherwise zero. Dependent variables for M2 is equal to 1 if the individual had one or more publications during the past five years. Dependent variables for M3 is equal to 1 if the individual had one or more conference papers during the past five years. Dependent variables of models M4 and M5 which are linear regressions are number of publications and conference papers. Data for M1 are coming from SDR 2013. Data for M2-M5 are SDR 2008 (the latest survey that asked publication and conference paper questions). Datapoints include all PhD holders in BSSR, whether or not employed in academia.
