# Supplementary material for: Recent trends in the U.S. Behavioral and Social Sciences Research (BSSR) workforce
Source: PLoS One. 2017 Feb 6;12(2):e0170887. doi: 10.1371/journal.pone.0170887 (PMC5293198; doi:10.1371/journal.pone.0170887)
Supplement: S3 Appendix — A2 Table shows full results of Table 6. (DOCX) [file pone.0170887.s003.docx]

**S3 Appendix. Complete Version of Table 6.**

Table A2 presents a complete version of Table 6 of the paper with all control variables.

**Table A2. Associations between demographic variables and the likelihood of receiving government funding among BSSR scientists employed in tenure-track or tenured positions- complete table.**

|  | Likelihood of receiving government funding | | | | |
| --- | --- | --- | --- | --- | --- |
| VARIABLES | All BSSR | Psychology | Economics | Political Sci | Sociology |
| Female | 0.85 | 0.88 | 1.13 | 1.13 | 0.83 |
|  | (0.09) | (0.14) | (0.37) | (0.40) | (0.24) |
| Race: (Ref: White) |  |  |  |  |  |
| Asian | 0.55*** | 0.79 | 0.54 | 0.58 | 0.26** |
|  | (0.10) | (0.23) | (0.21) | (0.31) | (0.14) |
| URM | 0.61*** | 0.49*** | 1.31 | 0.23*** | 0.55 |
|  | (0.08) | (0.10) | (0.43) | (0.13) | (0.21) |
| Profs: (Ref: Assistant Prof.) |  |  |  |  |  |
| Associate Prof. | 0.82 | 0.78 | 1.01 | 1.31 | 0.60 |
|  | (0.11) | (0.16) | (0.40) | (0.56) | (0.25) |
| Professor | 1.06 | 0.99 | 2.16* | 0.58 | 0.66 |
|  | (0.17) | (0.24) | (0.90) | (0.32) | (0.33) |
| Citizenship: US | 1.83*** | 2.29** | 3.08*** | 0.85 | 0.70 |
|  | (0.31) | (0.83) | (1.04) | (0.41) | (0.31) |
| Age | 0.99 | 1.00 | 1.00 | 1.02 | 0.97 |
|  | (0.01) | (0.01) | (0.02) | (0.02) | (0.02) |
| Marriage | 0.981 | 1.17 | 1.34 | 0.43 | 1.30 |
|  | (0.18) | (0.35) | (1.07) | (0.27) | (0.66) |
| Children | 1.13 | 0.90 | 1.83** | 1.78 | 0.89 |
|  | (0.12) | (0.15) | (0.51) | (0.66) | (0.29) |
| Spousework | 1.13 | 1.02 | 0.98 | 1.03 | 1.13 |
|  | (0.14) | (0.20) | (0.27) | (0.42) | (0.38) |
| Work Duration | 1.00 | 0.999 | 1.00 | 1.00 | 1.00* |
|  | (0.00) | (0.00) | (0.00) | (0.00) | (0.00) |
| Employer Size: (Ref: 1-99) |  |  |  |  |  |
| 100-4999 | 0.67 | 1.13 | 0.10** | 0.42** | 0.25*** |
|  | (0.34) | (0.91) | (0.10) | (0.16) | (0.10) |
| 5000+ | 2.02 | 3.50 | 0.30 |  |  |
|  | (1.02) | (2.75) | (0.29) |  |  |
| Major: (Ref: Psychology) |  |  |  |  |  |
| Economics | 0.57*** |  |  |  |  |
|  | (0.09) |  |  |  |  |
| Political science | 0.27*** |  |  |  |  |
|  | (0.05) |  |  |  |  |
| Sociology | 0.51*** |  |  |  |  |
|  | (0.08) |  |  |  |  |
| other social sciences | 0.52*** |  |  |  |  |
|  | (0.07) |  |  |  |  |
| Constant | 0.29* | 0.14* | 0.28 | 0.17 | 1.85 |
|  | (0.18) | (0.14) | (0.41) | (0.20) | (2.21) |
| Pseudo R^2^ | 0.09 | 0.07 | 0.13 | 0.08 | 0.08 |
| Observations | 2,841 | 882 | 538 | 433 | 364 |

*** p<0.01, ** p<0.05, * p<0.1

Notes: The table reports odds ratios. Standard errors are in parentheses. The models are logistic regressions, and the dependent variable is AnyGovFunding, which is equal to 1 if the individual received any federal funding during the past year and otherwise is equal to 0. Data are SDR 2013.
