# Supplementary material for: Recent trends in the U.S. Behavioral and Social Sciences Research (BSSR) workforce
Source: PLoS One. 2017 Feb 6;12(2):e0170887. doi: 10.1371/journal.pone.0170887 (PMC5293198; doi:10.1371/journal.pone.0170887)
Supplement: S4 Appendix — A3 and A4 Tables show full results of Tables 7 and 8. (DOCX) [file pone.0170887.s004.docx]

**S4 Appendix. Complete Version of Tables 7 and 8.**

A3 and A4 Tables present a complete version of Tables 7 and 8 of the paper reporting coefficients for all control variables.

**Table A3. Associations between demographic variables and publications among BSSR scientists employed in tenure-track or tenured positions - complete table.**

|  | Journal publications | | | | |
| --- | --- | --- | --- | --- | --- |
| VARIABLES | All BSSR | Psychology | Economics | Political Sci | Sociology |
| Female | -1.06*** | -1.09 | -1.53* | 0.52 | -1.97** |
|  | (0.39) | (0.89) | (0.82) | (0.64) | (0.92) |
| Race: (Ref: White) |  |  |  |  |  |
| Asian | -1.04 | -1.67 | -1.93* | -0.52 | -1.19 |
|  | (0.67) | (1.78) | (0.99) | (1.20) | (1.77) |
| URM | -1.60*** | -1.62 | -1.38 | -1.17 | -2.48** |
|  | (0.46) | (1.13) | (0.98) | (0.73) | (1.06) |
| Profs: (Ref: Assistant Prof.) |  |  |  |  |  |
| Associate Prof. | 2.40*** | 3.10*** | 3.93*** | 1.01 | 2.21* |
|  | (0.50) | (1.16) | (1.02) | (0.77) | (1.23) |
| Professor | 5.26*** | 7.25*** | 6.27*** | 3.23*** | 4.87*** |
|  | (0.59) | (1.40) | (1.15) | (0.89) | (1.46) |
| Citizenship: US | -1.46** | -3.40 | -2.08** | -0.50 | -0.81 |
|  | (0.73) | (2.90) | (1.02) | (1.21) | (1.83) |
| Major: (Ref: Psychology) |  |  |  |  |  |
| Economics | -3.45*** |  |  |  |  |
|  | (0.56) |  |  |  |  |
| Political science | -4.82*** |  |  |  |  |
|  | (0.53) |  |  |  |  |
| Sociology | -2.82*** |  |  |  |  |
|  | (0.56) |  |  |  |  |
| other social sciences | -3.22*** |  |  |  |  |
|  | (0.51) |  |  |  |  |
| Age | -0.09*** | -0.13** | -0.10** | -0.07** | 0.02 |
|  | (0.02) | (0.057) | (0.05) | (0.04) | (0.06) |
| Marriage | 0.41 | 1.12 | 0.58 | 1.60 | -0.04 |
|  | (0.68) | (1.60) | (1.72) | (1.11) | (1.50) |
| Children | 0.47 | 0.45 | 0.49 | -0.23 | 2.52** |
|  | (0.39) | (0.91) | (0.70) | (0.61) | (0.97) |
| Spousework | 0.54 | 1.13 | 0.072 | -0.34 | 0.66 |
|  | (0.44) | (1.12) | (0.74) | (0.69) | (1.10) |
| Work Duration | -0.007*** | -0.006 | -0.011*** | -0.005* | -0.01** |
|  | (0.002) | (0.004) | (0.003) | (0.003) | (0.005) |
| Employer Size: (Ref: 1-99) |  |  |  |  |  |
| 100-4999 | 2.54 | 4.38 | -1.86 | 2.85 | 1.73 |
|  | (2.22) | (4.16) | (4.53) | (4.00) | (5.73) |
| 5000+ | 5.49** | 8.63** | -0.66 | 4.31 | 5.35 |
|  | (2.20) | (4.12) | (4.52) | (3.98) | (5.68) |
| Constant | 7.74*** | 6.50 | 10.70** | 2.11 | -0.02 |
|  | (2.58) | (5.72) | (4.84) | (4.36) | (6.92) |
| R^2^ | 0.10 | 0.06 | 0.10 | 0.04 | 0.11 |
| Observations | 2,296 | 769 | 364 | 385 | 329 |

*** p<0.01, ** p<0.05, * p<0.1

**Table A4. Associations between demographic variables and conference papers among BSSR scientists employed in tenure-track or tenured positions - complete table.**

|  | Conference papers | | | | |
| --- | --- | --- | --- | --- | --- |
| VARIABLES | All BSSR | Psychology | Economics | Political Sci | Sociology |
| Female | -0.48 | 0.24 | -0.59 | 0.50 | -3.29** |
|  | (0.53) | (1.11) | (1.31) | (1.10) | (1.46) |
| Race: (Ref: White) |  |  |  |  |  |
| Asian | -2.10** | -2.07 | -3.17** | -1.29 | -4.39 |
|  | (0.92) | (2.23) | (1.58) | (2.07) | (2.81) |
| URM | -2.31*** | -3.64** | -0.66 | -2.94** | -3.61** |
|  | (0.63) | (1.41) | (1.57) | (1.26) | (1.68) |
| Profs: (Ref: Assistant Prof.) |  |  |  |  |  |
| Associate Prof. | 2.63*** | 4.53*** | 1.43 | 0.71 | 2.91 |
|  | (0.69) | (1.45) | (1.63) | (1.33) | (1.95) |
| Professor | 6.02*** | 9.29*** | 6.03*** | 3.28** | 6.00** |
|  | (0.82) | (1.76) | (1.83) | (1.54) | (2.33) |
| Citizenship: US | -4.09*** | -10.07*** | -3.78** | -6.05*** | -2.24 |
|  | (1.01) | (3.64) | (1.63) | (2.09) | (2.91) |
| Major: (Ref: Psychology) |  |  |  |  |  |
| Economics | -4.34*** |  |  |  |  |
|  | (0.78) |  |  |  |  |
| Political science | -4.58*** |  |  |  |  |
|  | (0.74) |  |  |  |  |
| Sociology | -2.24*** |  |  |  |  |
|  | (0.77) |  |  |  |  |
| other social sciences | -2.65*** |  |  |  |  |
|  | (0.70) |  |  |  |  |
| Age | -0.17*** | -0.33*** | -0.11 | -0.14** | -0.02 |
|  | (0.03) | (0.07) | (0.08) | (0.06) | (0.09) |
| Marriage | -0.35 | 1.41 | -0.20 | -2.16 | -0.22 |
|  | (0.94) | (2.00) | (2.75) | (1.92) | (2.39) |
| Children | 0.21 | 0.02 | -0.71 | 0.50 | 2.20 |
|  | (0.54) | (1.14) | (1.12) | (1.06) | (1.55) |
| Spousework | 0.35 | -0.34 | 0.44 | -0.42 | 1.11 |
|  | (0.61) | (1.41) | (1.19) | (1.19) | (1.75) |
| Work Duration | -0.007** | -0.001 | -0.012** | -0.006 | -0.016** |
|  | (0.003) | (0.005) | (0.006) | (0.005) | (0.007) |
| Employer Size: (Ref: 1-99) |  |  |  |  |  |
| 100-4999 | 3.14 | 3.64 | 2.27 | 8.80 | -1.42 |
|  | (3.06) | (5.22) | (7.23) | (6.90) | (9.11) |
| 5000+ | 6.60** | 8.67* | 3.74 | 10.52 | 2.23 |
|  | (3.04) | (5.17) | (7.21) | (6.87) | (9.02) |
| Constant | 17.70*** | 25.95*** | 13.88* | 12.67* | 12.06 |
|  | (3.57) | (7.17) | (7.73) | (7.53) | (10.99) |
| R^2^ | 0.07 | 0.07 | 0.04 | 0.05 | 0.05 |
| Observations | 2,296 | 769 | 364 | 385 | 329 |

*** p<0.01, ** p<0.05, * p<0.1

Notes: Standard errors are in parentheses. The models are linear regressions. The dependent variables are number of publications and number of conference papers during the past five years. Data from SDR 2008 (the latest survey that asked publication and conference paper questions); only individuals employed in tenure-track or tenured positions in academia.
